# Genomic and prognostic heterogeneity among *RAS/BRAF* ^V600E^/*TP53* co‐mutated resectable colorectal liver metastases

**DOI:** 10.1002/1878-0261.12885

**Published:** 2021-01-08

**Authors:** Kaja C. G. Berg, Tuva H. Brunsell, Anita Sveen, Sharmini Alagaratnam, Merete Bjørnslett, Merete Hektoen, Kristoffer W. Brudvik, Bård I. Røsok, Bjørn Atle Bjørnbeth, Arild Nesbakken, Ragnhild A. Lothe

**Affiliations:** ^1^ Department of Molecular Oncology Institute for Cancer Research Oslo University Hospital Norway; ^2^ K.G.Jebsen Colorectal Cancer Research Centre Division for Cancer Medicine Oslo University Hospital Norway; ^3^ Institute for Clinical Medicine Faculty of Medicine University of Oslo Norway; ^4^ Department of Gastrointestinal Surgery Oslo University Hospital Norway

**Keywords:** colorectal liver metastases, DNA copy number aberrations, gene mutations, tumor heterogeneity

## Abstract

Hepatic resection is potentially curative for patients with colorectal liver metastases, but the treatment benefit varies. *KRAS*/*NRAS* (*RAS)*/*TP53* co‐mutations are associated with a poor prognosis after resection, but there is large variation in patient outcome within the mutation groups, and genetic testing is currently not used to evaluate benefit from surgery. We have investigated the potential for improved prognostic stratification by combined biomarker analysis with DNA copy number aberrations (CNAs), and taking tumor heterogeneity into account. We determined the mutation status of *RAS*, *BRAF* ^V600^, and *TP53* in 441 liver lesions from 171 patients treated by partial hepatectomy for metastatic colorectal cancer. CNAs were profiled in 232 tumors from 67 of the patients. Mutations and high‐level amplifications of cancer‐critical genes, the latter including *ERBB2* and *EGFR*, were predominantly homogeneous within patients. *RAS*/*BRAF* ^V600E^ and *TP53* co‐mutations were associated with a poor patient outcome (hazard ratio, HR, 3.9, 95% confidence interval, CI, 1.3–11.1, *P* = 0.012) in multivariable analyses with clinicopathological variables. The genome‐wide CNA burden and intrapatient intermetastatic CNA heterogeneity varied within the mutation groups, and the CNA burden had prognostic associations in univariable analysis. Combined prognostic analyses of *RAS*/*BRAF* ^V600E^/*TP53* mutations and CNAs, either as a high CNA burden or high intermetastatic CNA heterogeneity, identified patients with a particularly poor outcome (co‐mutation/high CNA burden: HR 2.7, 95% CI 1.2–5.9, *P* = 0.013; co‐mutation/high CNA heterogeneity: HR 2.5, 95% CI 1.1–5.6, *P* = 0.022). In conclusion, DNA copy number profiling identified genomic and prognostic heterogeneity among patients with resectable colorectal liver metastases with co‐mutated *RAS*/*BRAF* ^V600E^/*TP53*.

## Graphical abstract

Colorectal cancer commonly metastasizes into multiple liver foci, but intermetastatic heterogeneity remains poorly described. Here, we demonstrate that mutations of *RAS/BRAF/TP53* are homogeneous within patients, while DNA copy number aberrations can vary greatly. *RAS/BRAF/TP53* co‐mutations conferred a dismal prognosis, and co‐mutations combined with a high burden and heterogeneity of copy number aberrations identified patients with the poorest outcome.

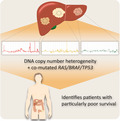

**Abbreviations**
5y‐CSS: five‐year cancer‐specific survival
CNA: copy number aberrations
CRC: colorectal cancer
CRLM: colorectal liver metastases
MSI: microsatellite instable
MSS: microsatellite stable

## 1 Introduction

Approximately 30% of all colorectal cancer (CRC) patients develop metastases to the liver during their disease course, of whom 20% undergo hepatic resection as a potentially curable treatment [1, 2]. In a Norwegian study, the five‐year overall and disease‐free survival was 46% and 24%, respectively, after partial hepatectomy [3], compared to a 5‐year relative survival rate of 15–22% for patients with distant metastases from CRC overall [4]. Around one third of the patients experience early recurrence following resection [3, 5, 6], and there are currently no strong markers for prediction of long‐term benefit from surgery [7, 8].

Mutations in *RAS (KRAS* and *NRAS)* have consistently been associated with a poor prognosis among patients with resectable colorectal liver metastases (CRLM) [9, 10, 11], and it has been suggested that surgical treatment is less beneficial in patients with *RAS* mutations [12]. However, the prognostic effect size is modest [13] and it was recently proposed that the effect is limited to tumors with co‐occurring *TP53* mutations [14, 15], or co‐occurring *TP53* and *SMAD4* mutations [16]. *BRAF* ^V600E^ mutations have a stronger prognostic effect size, but the prognostic value is limited by the low prevalence of this marker among patients with resectable CRLM [17].

Colorectal liver metastases commonly present with multiple distinct liver lesions. Cancer‐critical genes with a high mutation prevalence in CRC generally have a homogeneous mutation pattern across metastatic lesions from the same patient [18, 19], although treatment pressure may cause subclonal expansion, as illustrated by the emergence of resistant subclones with pre‐existing or acquired *KRAS* mutations during anti‐EGFR therapy [20, 21]. More extensive mutation heterogeneity has been demonstrated in other protein‐coding genes, both in intratumor and intertumor comparisons [22, 23, 24]. We have previously shown that there is considerable intrapatient intermetastatic heterogeneity also on the DNA copy number level [25]. The clinical impact of such intermetastatic molecular heterogeneity remains poorly defined [22, 23, 26], although our study suggested that a high degree of heterogeneity of DNA copy number aberrations (CNAs) is associated with a poor prognosis [25]. We have previously also reported differential radiological responses to standard neoadjuvant treatment among metastatic lesions in a subgroup of approximately 10% of patients with resectable CRLM [27]. How this phenotypic heterogeneity relates to molecular heterogeneity is currently not clear, but the poor survival rate of this patient subgroup after surgery highlights the potential clinical importance of intermetastatic heterogeneity.

Here, we performed combined biomarker analyses in relation to outcome among patients with resectable CRLM, taking tumor heterogeneity into account. We investigated mutations in *KRAS, NRAS, BRAF* ^V600E^, and *TP53*, combined with the overall burden and intermetastatic heterogeneity of CNAs.

## 2 Methods

### 2.1 Patient samples

The study included fresh‐frozen samples of 460 liver metastases from 176 patients who underwent resection for CRLM at Oslo University Hospital, Oslo, Norway, between October 2013 and February 2018. All patients provided signed informed consents, and the study was conducted in line with the Helsinki declaration with approval by the Norwegian Data Protection Authority and the Regional Committee for Medical and Health Research Ethics, South‐Eastern Norway (ref no.: 1.2005.1629;2010/1805).

Fresh‐frozen tumor tissue samples (15–30 mg) were homogenized in liquid nitrogen and DNA was extracted using the AllPrep Universal DNA/RNA/miRNA protocol (Qiagen, Hilden, Germany). DNA quality and concentrations were assessed by NanoDrop 1000 spectrophotometer (version 3.7.1, Thermo Fisher Scientific, Waltham, MA, USA) and Qubit fluorometer (Thermo Fischer Scientific).

**Fig. 1:**
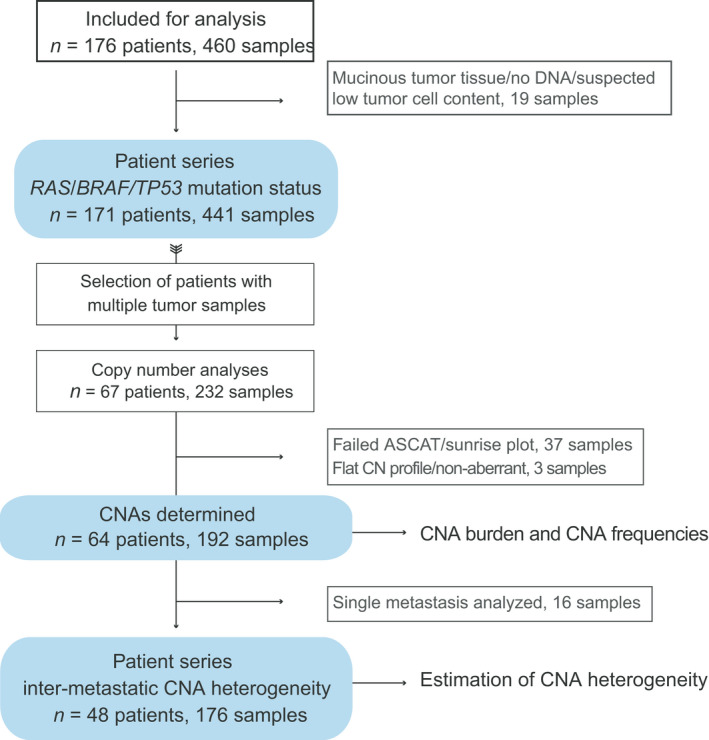
Overview of the included patients and samples in the study.

Five patients were excluded from analyses due to mucinous tumor tissue, poor DNA quality, or suspicion of low tumor cell content. In total, 441 liver metastases from 171 patients were included for mutation analyses (Fig. 1), of which 102 patients had multiple lesions analyzed (median of 3 metastatic lesions per patient, range 2–9).

### 2.2 Mutation and microsatellite instability analyses

A total of 355 metastatic tumor samples from 103 patients have previously been analyzed for hotspot mutations in *BRAF* exon 15 and *KRAS* and *NRAS* exons 2–4 by Sanger sequencing [19]. The remaining 86 tumor samples and 68 patients were analyzed in the present study.

All 441 tumor samples were also sequenced for all coding regions of *TP53* (exons 2–11). In summary, three singleplex PCR reactions were used to analyze *TP53* exons 2–4, 5–6, and 7–9, respectively, by amplifying 50 ng of DNA in a reaction mix containing 10× HotStar‐buffer, dNTP, HotStar Taq polymerase (Qiagen), and the primers described in Table S1. *TP53* exons 10 and 11 were analyzed in a separate multiplex PCR reaction by amplification of 50 ng of DNA using the 2× Multiplex PCR kit (Qiagen). PCR products were purified using Illustra ExoProStar 1‐step (GE Healthcare, Chicago, IL, USA), and the Applied Biosystems BigDye Terminator v1.1 Cycle Sequencing Kit and Applied Biosystems 3730 DNA Analyzer were used for sequencing (both Thermo Fisher Scientific). DNA from the blood of two healthy donors was used as controls. The results were analyzed using Applied Biosystems Sequencing Analysis software v5.3.1 and SeqScape software v2.5 (Thermo Fisher Scientific) and scored independently by two investigators. Synonymous mutations were not reported. All mutations and cases of intrapatient mutation heterogeneity were validated in independent PCR reactions, some also with ultra‐deep targeted sequencing with the Illumina TruSight Tumor 15 gene panel as described in [19].

All tumors were analyzed for microsatellite instability (MSI) status using PCR‐based marker analyses, either as previously described using BAT25/BAT26 [28], or using the five markers incorporated in the MSI Analysis System version 1.2 (Promega, Fitchburg, WI, USA). Uncertain cases after analyses of BAT25/BAT26 were re‐analyzed with the MSI Analysis System.

### 2.3 DNA copy number analyses

A total of 232 lesions from the first 67 patients with multiple metastases sampled were analyzed by genome‐wide DNA copy number profiling using the Applied Biosystems CytoScanHD array (Thermo Fisher Scientific). The procedure was conducted according to the manufacturer’s instructions, following the CytoScan Assay Manual Protocol. Resulting raw‐intensity CEL files were preprocessed with the R package rawcopy (v1.1) [29], and subsequently segmented by ascat (v2.5) [30], with penalty parameter set to 25 and chromosomes X and Y excluded. A primary interest was to estimate the level of CNA heterogeneity among samples from the same patient. This estimate is highly sensitive to poor data quality, and strict quality control was therefore performed on the segmented data by careful visual inspection of copy number profiles and Sunrise plots produced by ASCAT. Samples with nonaberrant profiles (no/few CNAs) or poor Sunrise plots were excluded, retaining 192 lesions from 64 patients for further analyses. Copy number gain and loss was called for segments with ≥ 1 or ≤ −1 copies relative to the median genome‐wide copy number estimated by ASCAT, respectively. For comparison with the data processing approach used in our previous study [25], the preprocessed data from rawcopy was additionally segmented by the PCF algorithm implemented in the R package copynumber [31] with the penalty parameter (gamma) set to 100.

To enable analyses across samples, the sample‐wise segmented data were further split into their smallest genomic regions of overlap by computationally introducing breakpoints at every unique breakpoint occurring in any sample in the total dataset.

The COSMIC Cancer Gene Census version 86 [32] was used to define cancer‐critical genes, (both Tier 1 and Tier 2 genes considered). Of the 719 genes, 672 were covered in the CNA data.

A sample‐wise estimate of the overall CNA burden was calculated as the fraction of the genome (per cent of base pairs) with aberrant copy number. For patients with multiple lesions, the mean CNA burden was used for patient‐wise analyses. Estimates of ploidy were derived from ASCAT.

Unpublished DNA copy number data were available for three matching primary tumors for comparison of amplification status in the metastases.

### 2.4 Estimation of intrapatient intermetastatic copy number heterogeneity

Intrapatient intermetastatic DNA copy number heterogeneity was analyzed by three different approaches. First, the genome‐wide matrix of estimated copy numbers was used to perform pairwise comparisons among metastatic lesions from each patient based on Euclidean distances, using the *dist* function implemented in the R stats package. To obtain one heterogeneity measure per patient, the mean Euclidean distance of all pairwise comparisons was calculated, in accordance with the approach used in our previous study [25]. Second, the pairwise distance was calculated as in the first approach but using Pearson correlation‐based distance. Third, CNA heterogeneity was assessed by a gene‐wise estimation (protein‐coding genes from UCSC known genes) of the fraction of CNAs within a patient that were not common across the lesions, that is, genes with aberrant copy number in one or more lesions but not in all. The heterogeneity calling was more conservative with this approach, as only events exceeding the copy number gain/loss thresholds were considered heterogeneous, while genes consistently affected by gain (or loss) but with varying amplitudes were regarded as homogeneous CNA events.

The patient‐wise CNA heterogeneity measure was categorized as high or low relative to the median across the patients.

For robustness, data segmented with the PCF algorithm were used to estimate copy number heterogeneity (distance‐based) in the same manner as in our previous study [25], by calculating the average pairwise Euclidean distance between DNA segments with a variance of > 0.03 among samples from each patient. The distance measured obtained from ASCAT and PCF showed good correlation (Spearman’s rho 0.58, *P* < 0.001; Fig. S1a).

### 2.5 Statistical analyses

Pairwise comparisons of variables between groups were done by nonparametric Wilcoxon rank‐sum tests for continuous variables and with Fisher’s exact test for categorical data, both implemented in the R stats package.

Survival analyses were performed with 5‐year cancer‐specific survival (5y‐CSS) as the end point. Time to death from CRC was measured from start of treatment of the liver metastases (either neoadjuvant systemic treatment or surgery), and deaths from other causes were censored [33]. Only patients with MSS cancers and R0 or R1 status in the liver after resection were included in survival analyses (*n* = 165 of 171 patients in the full cohort, *n* = 62 of 64 patients in CNA burden analyses, *n* = 46 of 48 in CNA heterogeneity analyses). Kaplan–Meier estimates and log rank tests were used for comparisons of variables with only two groups, using the *survdiff* function in the R survival package. For comparisons of more than two groups, log rank tests for trend were performed using the *comp* function in the R survMisc package. All Kaplan–Meier plots were made with the *ggsurvplot* function in the R survminer package. Univariable and multivariable Cox regression analyses were performed with the *coxph* function in the R survival package. *P* values were not adjusted for multiple testing. The prognostic markers evaluated (mutations in *KRAS*, *NRAS*, *BRAF,* and *TP53,* as well as the two CNA measures) were predetermined based on previous work; however, the size of the study population was determined based on availability of material, and the study was therefore exploratory.

## 3 Results

### 3.1 Concordant driver gene mutation profiles among multiple resected CRLM

**Fig. 2:**
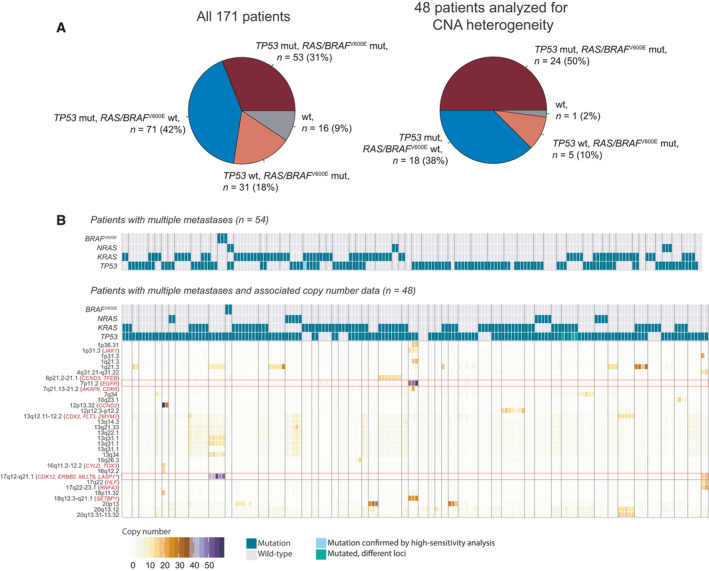
(A) Prevalence of *RAS*/*BRAF* ^V600E^ mutation only, *TP53* mutation only, and co‐mutation of *RAS/BRAF* ^V600E^/*TP53* in the full cohort (*n* = 171) and in the subset of patients with associated DNA copy number data from multiple lesions (*n* = 48). (B) The upper panel shows patients with multiple metastases that were analyzed by sequencing only (*n* = 54 patients). The lower panel shows patients with multiple metastases analyzed for both mutations and CNAs (*n* = 48 patients), and only lesions with good quality CNA data from the same resection were included. Vertical gray lines separate each patient. Cancer‐critical genes are marked in red writing and the red horizontal boxes highlight the therapeutically relevant targets *EGFR* and *ERBB2*. The mutation status was the same in all metastatic deposits analyzed from each patient, with the exception of *TP53* in four patients. One patient had *TP53* mutations at two different loci among the lesions (pale green), and three patients had unavailable high‐sensitivity sequencing data to rule out heterogeneity. Both mutations in the driver genes *BRAF*, *NRAS*, *KRAS,* and *TP53*, as well as high‐level amplifications (> 15 additional copies), were predominantly homogeneous within patients. *MLLT6 and *LASP1*: only amplified in the patient to the far right of the heatmap.

Among resected CRLM from 171 patients, the patient‐wise mutation prevalence was 42.7% (73/171) for *KRAS*, 4.7% (8/171) for *NRAS*, 1.8% (3/171) for *BRAF* ^V600E^ and 72.5% (124/171) for *TP53*. *KRAS*, *NRAS,* and *BRAF* ^V600E^ mutations were mutually exclusive, while *RAS*/*BRAF* ^V600E^ co‐occurred with TP53 mutations in 31% of the patients (Fig. 2A,B). The mutation status of the four genes was homogeneous in all metastatic deposits from each patient when ultra‐deep targeted sequencing was applied; however, three patients had unconfirmed heterogeneity (Fig. 2B) due to the lack of high‐sensitivity data. Another patient had intermetastatic heterogeneity in the specific loci affected by *TP53* mutation and displayed p. Asp184fs mutations in two lesions and p. Arg273His mutations in three lesions (all five lesions had the same *KRAS* mutation). These patients were classified as mutated.

All patients except one (99%) had microsatellite stable (MSS) tumors. DNA copy number profiling indicated larger intrapatient intermetastatic variation in the sequenced genes, and heterogeneous DNA copy number status was found in 16/48 patients (33%) for *BRAF*, 24/48 patients (50%) for *KRAS*, 19/48 patients (40%) for *NRAS*, and 9/48 (19%) for *TP53* (Fig. S1b). However, this was associated with a larger genome‐wide level of CNA heterogeneity in the same patients (Fig. S1c), indicating that these four genes were not specifically targeted.

None of the four genes had any high‐level amplifications events (≤ 6 additional copies), but a genome‐wide search identified high‐level amplifications (≥ 15 additional copies) in CRLM from 22 (34%) of the 64 patients analyzed. Among cancer‐critical genes (defined in the COSMIC Cancer Gene Census), recurrent high‐level amplifications were found only of *ERBB2* in two patients, while *EGFR* and the cell cycle genes *CDK6*, *CCND2*, and *CCND3* were amplified in one patient each (Table 1). Notably, none of the patients with *ERBB2* or *EGFR* amplifications had received anti‐EGFR therapy. Additionally, the nominated target *TOX3* [34] was amplified in one patient. The amplification events were commonly concordant in intrapatient intermetastatic comparisons, albeit with variation in the amplitude (Fig. 2B). Among patients with multiple metastases analyzed, 31% of high‐level amplifications were homogeneous, and an additional 38% of the amplifications had lower‐amplitude gains (≥ 5 additional copies) in all other metastases from the same patient. Corresponding numbers for amplification events affecting cancer‐critical genes were 50% and 25% (Fig. S2). Notably, intrapatient concordance was also found for the clinically relevant target genes *ERBB2* and *EGFR*, including in comparison with the primary tumor of one patient with *ERBB2* amplification (9 additional copies in the primary and 17 and 18 additional copies in the two metastases). For the patient with two CRLM with *CCND2* amplifications (29 and 47 additional copies), the primary tumor had 32 additional copies of this gene. The patient with *CCND3* amplification in the range of 14–16 additional copies in all 7 metastases did not have a detectable *CCND3* amplification in the primary tumor.

**Table 1:** Intermetastatic heterogeneity status for high‐level amplifications of cancer‐critical genes.

Patient	Number of tumors analyzed	Region (hg19)	Cancer‐critical genes in region	Copy number (range among tumors)^a^	Intrapatient intermetastatic heterogeneity
1	7	chr6:39863162‐42671542	*CCND3, TFEB*	14–16^b^	No (when also counting intermediate‐level amplifications of 14 copies)
2	3	chr1:65183880‐66527443	*JAK1*	10–15	No (when also counting intermediate‐level amplifications of 10–14 copies)
		chr7:54576560‐56118007	*EGFR*	37–58	No
		chr7:90792390‐92573683	*AKAP9, CDK6*	0–22	Yes
		chr18:41497284‐42716881	*SETBP1*	18–20	No
3	2	chr12:4279446‐4431071	*CCND2*	29–47^c^	No
		chr16:40873444‐53153010	*CYLD, TOX3* ^d^	0–16^c^	Yes
4	6	chr13:20528021‐21570265	*ZMYM2*	3–15	Yes
		chr13:28302602‐28662578	*CDX2*, *FLT3*	3–15	Yes
5	5	chr17:37604254‐37701703	*CDK12*	16–41	No
		chr17:37704051‐38191836	*ERBB2*	41–55	No
6	2	chr17:36841569‐37669141	*LASP1, MLLT6*	12–18^e^	No (when also counting intermediate‐level amplifications of 12–14 copies)
		chr17:37669142‐37993556	*CDK12, ERBB2*	17–18^e^	No
		chr17:53268056‐53593625	*HLF*	14–18^e^	No (when also counting intermediate‐level amplifications of 14 copies)
		chr17:56250122‐57541594	*RNF43*	14–18^e^	No (when also counting intermediate‐level amplifications of 14 copies)

^a^ Number of additional copies, relative to the estimated ploidy.

^b^ Primary tumor: no amplification.

^c^ Primary tumor: 32 copies of *CCDN2* and a neutral copy number state for *CYLD* and *TOX3*.

^d^ Not a COSMIC gene.

^e^ Primary tumor: 7 copies of *MLLT6*, 9 copies of LASP1, *CDK12*, *ERBB2*, 7 copies of *HLF* and 8 copies of *RNF43*.

### 3.2 Frequent intermetastatic DNA copy number heterogeneity on the genome‐wide scale

The genome‐wide CNA frequencies, summarized patient‐wise, were in accordance with the well‐known aberration profiles of CRC (among 192 lesions from 64 patients; Fig. S3). Frequent copy number gains were found on chromosome arms 7p and q, 8q, 13q, and 20q, and copy number losses on 1p, 4p and q, 8p, 17p, and 18p and q.

**Fig. 3:**
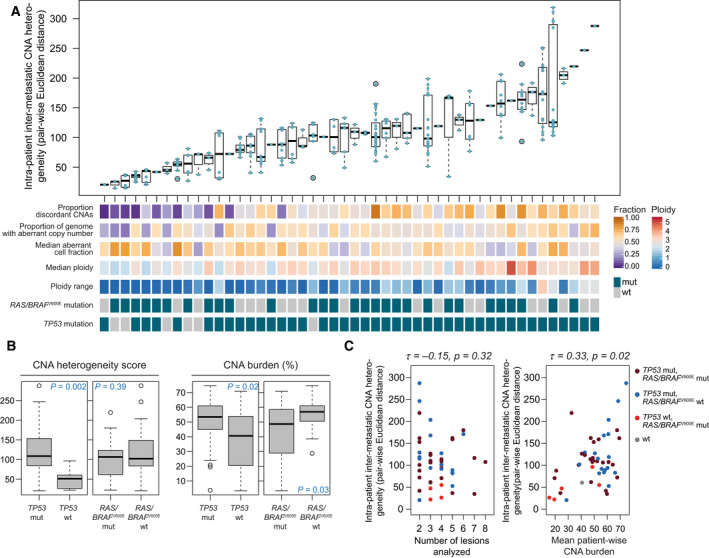
(A) Genomic characteristics of 48 patients analyzed for DNA copy number heterogeneity. Top: Pairwise Euclidean distance measures ranged between 21 and 319, and heterogeneity scores per patient (mean pairwise distance measure per patient) ranged from 21 to 287 (median 104). Bottom: the bars indicate the fraction of CNAs found in one or more metastatic lesions but not all (discordant CNAs), the patient‐wise average CNA burden (proportion of the genome with aberrant copy number), the patient‐wise median and range of ploidy states among the metastases, and *RAS*/*BRAF* ^V600E^ and *TP53* mutation status. (B) CNA heterogeneity was significantly associated with *TP53*, but not *RAS*/BRAF^V600E^ mutation status (*n* = 42/*n* = 6 *TP53* mutated/wild‐type; *n* = 29/*n* = 19 *RAS*/*BRAF* ^V600E^ mutated/wild‐type). *TP53* mutation was also associated with higher CNA burden, while *RAS*/*BRAF* ^V600E^ mutations were associated with lower CNA burden (*n* = 51/*n* = 13 *TP53* mutated/wild‐type; *n* = 42/*n* = 22 *RAS*/*BRAF* ^V600E^ mutated/wild‐type). The CNA estimates still varied within the mutational subgroups, with interquartile range between 27 and 65 for CNA heterogeneity (Euclidean distance) and 10–33 for CNA burden (%). (C) CNA heterogeneity assessed as the mean Euclidean distance was not correlated with the number of lesions analyzed, and only weakly to the overall CNA burden.

High‐quality DNA copy number data were available for at least two metastatic lesions from 48 patients (Fig. 1), including a total of 176 tumors and a median of 4 tumors per patient (range 2–8). For these patients, intermetastatic CNA heterogeneity was estimated by three different approaches (Methods) and with three different sets of input data of varying width of genomic coverage (across the whole genome, from protein‐coding genes, or from only the subset of 672 cancer‐critical genes). The different estimates were strongly correlated, indicating robustness to both the approach (Spearman’s rho ≥ 0.63, *P* < 0.001) and to the width of genomic coverage (Spearman’s rho ≥ 0.93, *P* < 0.001; Fig. S4). Further analyses were performed using the genome‐wide Euclidean distance‐derived heterogeneity measure, consistent with our previous study [25]. There was a large variation among patients in the degree of intermetastatic CNA heterogeneity (Fig. 3A). This CNA heterogeneity was independent of the number of lesions analyzed per patient, the patient‐wise median aberrant cell fraction and the *RAS*/*BRAF* ^V600E^ mutation status, but was correlated with the patient‐wise median ploidy state and ploidy range, and the *TP53* mutation status (Fig. 3B,C; Table S2). The CNA heterogeneity score was also weakly correlated with the mean patient‐wise CNA burden of the metastases (analyzed as the fraction of the genome with aberrant copy numbers; Spearman’s rho 0.33, *P = *0.02; Fig. 3C).

### 3.3 Co‐mutated *RAS*/*BRAF* ^V600E^ and *TP53* are associated with poor patient outcome

The 165 patients with MSS cancers that were treated with R0 or R1 hepatic resection had a median cancer‐specific survival of 48 months and a 5y‐CSS rate of 40%. The 139 patients with R0 or R1 status overall had a median cancer‐specific survival of 50 months and a 5y‐CSS rate of 44%. Several clinicopathological factors (Table 2) were associated with poor patient outcome in univariable Cox regression analysis, and gender, size of the largest metastasis, R‐status in the liver and presence of extrahepatic disease remained significant in multivariable analyses (Table 3).

**Table 2:** Clinicopathological characteristics of all 171 patients and 48 patients with multiple metastases and associated CNA data.

Variable	Total patient series, *n* = 171		Subset for copy number heterogeneity analyses, *n* = 48	
	*n* (range)	%	*n* (range)	%
Age at surgery, median (range)	66 (21–85)	–	67 (21–85)	–
Male sex	106	62	34	71
Primary tumor in right colon^a^	36	21	12	25
Positive nodal status primary	116^b^	68	28	58
Synchronous liver metastases^c^	134	78	39	81
Previous resection of CRLM	37	22	9	19
Previous chemotherapy	52	30	9	19
Chemotherapy for these CRLM	131	77	43	90
Targeted agents for these CRLM	47	27	17	35
Median (range) number of chemotherapy cycles	4 (1–41)	–	5 (1–41)	–
Median (range) size largest CRLM, mm^d^	27 (6–120)	–	29 (10–113)	–
Median (range) number of CRLM^d^	4 (1–23)	–	6 (1–20)	–
Median (range) number of analyzed CRLM	2 (1–9)	–	4 (2–8)	–
Laparoscopic procedure	39	23	3	6
Two‐stage hepatectomy	33	19	18	38
Radiofrequency ablation	23	13	4	8
R‐status liver				
R0‐resection	71	42	14	29
R1‐resection^e^	95	56	32	67
R2‐resection^f^	5^g^	3	2^h^	4
Extrahepatic disease (%)	32	19	10	21

^a^ Including the transverse colon.

^b^ Missing data for six patients.

^c^ First liver metastases detected within 6 months of primary tumor diagnosis.

^d^ On radiologic imaging before treatment.

^e^ < 1 mm margin or RFA treatment.

^f^ Not completed second‐stage hepatectomy due to disease progression in observation period (*n* = 2) and missing lesions after neoadjuvant chemotherapy (*n* = 3).

^g^ Two patients with R2‐resection of the liver also had extrahepatic disease.

^h^ One patient with R2‐resection of the liver also had extrahepatic disease.

**Table 3:** Cox regression analyses.

Variable		Univariable analysis		Multivariable analysis		*N* patients (events)
		HR^a^ (95% CI^b^)	*P*‐value	HR^a^ (95% CI^b^)	*P*‐value	
Age at surgery > cohort median		1.2 (0.8–1.8)	0.444			165 (92)
Male sex		2.7 (1.7–4.3)	**< 0.001**	2.7 (1.7–4.4)	**< 0.001**	
Primary tumor in right colon		1.4 (0.8–2.2)	0.213			
Positive nodal status primary		0.9 (0.6–1.4)	0.617			
Synchronous liver metastases		0.8 (0.5–1.2)	0.269			
Previous resection of CRLM		0.6 (0.4–1.1)	0.094			
Previous chemotherapy		1.2 (0.8–1.9)	0.356			
Chemotherapy for these CRLM		1.4 (0.9–2.4)	0.169			
Targeted agents for these CRLM		0.9 (0.6–1.4)	0.665			
Number of cycles > cohort median		1.6 (1.1–2.5)	**0.018**	1.4 (0.9–2.1)	0.168	
Size largest CRLM, mm > cohort median		1.7 (1.1–2.6)	**0.010**	1.6 (1.1–2.5)	**0.026**	
Single metastasis		0.6 (0.4–1.1)	0.124			
Number of CRLM > cohort median^c^		1.2 (0.8–1.9)	0.328			
Laparoscopic procedure		0.8 (0.5–1.3)	0.303			
Two‐stage hepatectomy		1.3 (0.8–2.1)	0.236			
Radiofrequency ablation		0.9 (0.5–1.7)	0.778			
R‐status liver^d^		1.6 (1.0–2.4)	**0.034**	1.7 (1.1–2.7)	**0.013**	
Extrahepatic disease		2.7 (1.7–4.3)	**< 0.001**	2.2 (1.3–3.6)	**0.003**	
RAS/BRAF^V600E^ and TP53 co‐mutation yes/no		1.9 (1.2–2.9)	**0.003**			
RAS/BRAF^V600E^ and TP53 co‐mutation^e^	TP53 only	2.3 (0.8–6.6)	0.106	2.4 (0.9–6.9)	0.096	
	RAS/BRAF^V600E^ only	2.6 (0.9–7.8)	0.089	3.0 (1.0–9.0)	0.054	
	co‐mut	4.1 (1.5–11.6)	**0.007**	3.9 (1.3–11.1)	**0.012**	
RAS/BRAF^V600E^ and TP53 co‐mutation and high mean patient‐wise CNA burden^f^	Co‐mutation and low CNA burden	1.5 (0.7–3.2)	0.281			62 (40)
	Co‐mutation and high CNA burden	2.7 (1.2–5.9)	**0.013**			
RAS/BRAF^V600E^ and TP53 co‐mutation and high intermetastatic CNA heterogeneity^f^	Co‐mutation and low CNA heterogeneity	1.6 (0.6–4.5)	0.365			46 (30)
	Co‐mutation and high CNA heterogeneity	2.5 (1.1–5.6)	**0.022**			

*P*‐values significant on a 5% level are highlighted in bold.

^a^ Hazard ratio.

^b^ Confidence interval.

^c^ As seen on radiological evaluation (CT/MRI) before surgery.

^d^ R0 versus R1.

^e^ Reference group: co‐wt.

^f^ Reference group: no co‐mutation.

**Fig. 4:**
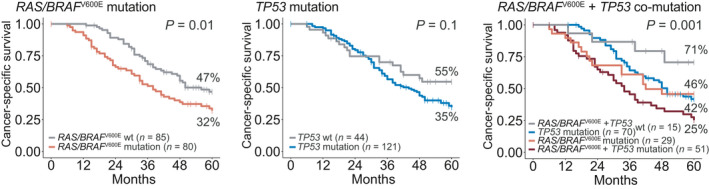
Five‐year CSS according to mutation status. *P* values are derived from log rank tests for comparisons of two groups and log rank tests for trend for comparisons of more than two groups. For pairwise comparisons, *RAS*/*BRAF* ^V600E^/*TP53* co‐mutation was associated with significantly worse survival than double wild‐type (*P* = 0.006) and *TP53* mutation only (*P* = 0.01), but not compared to *RAS*/*BRAF* ^V600E^ mutations only (*P* = 0.2). Wt = wild‐type.

*RAS*/*BRAF* ^V600E^ mutations, but not *TP53* mutations, were associated with a poor 5y‐CSS in univariable analyses (*RAS*/*BRAF* ^V600E^: 32% for mutated versus 47% for wild‐type, *P* = 0.01; *TP53:* 35% for mutated versus 55% for wild‐type, *P* = 0.1; Fig. 4). Co‐mutations of *RAS*/*BRAF* ^V600E^ and *TP53* had a strong prognostic impact, with a 5y‐CSS of 25%, compared to 46% for patients with *RAS*/*BRAF* ^V600E^ mutation only, 42% for *TP53* mutation only, and 71% in patients with wild‐type status for all four genes (*P* = 0.001, test for trend, Fig. 4). Co‐mutated *RAS*/*BRAF* ^V600E^ and *TP53* was not significant compared to patients with *RAS*/*BRAF* ^V600E^ mutations only (*P* = 0.2). The prognostic role of co‐mutations was not driven by patients with *BRAF* ^V600E^ mutations, as the analyses remained significant upon exclusion of 3 patients with BRAF^V600E^ mutations (Fig. S5a).

Co‐mutations of *RAS*/*BRAF* ^V600E^ and *TP53* were enriched in patients with a right‐sided primary tumor location and with extrahepatic metastases and depleted among patients with positive nodal status and those receiving neoadjuvant anti‐EGFR or VEGF treatment (Table S3). However, co‐mutation remained significant in multivariable analyses including clinicopathological factors (Table 3).

### 3.4 Genome‐wide CNA profiles have poor prognostic associations

**Fig. 5:**
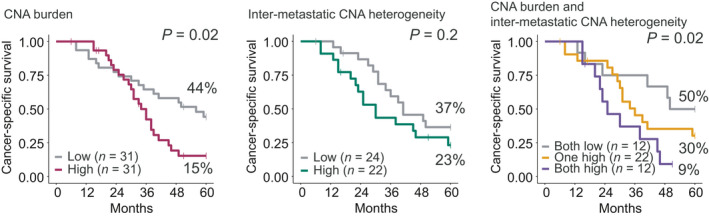
Five‐year CSS according to CNA burden (left), CNA heterogeneity (middle), and both measures combined (right). *P* values are derived from log rank tests for comparisons of two groups and log rank tests for trend for comparisons of more than two groups.

Two measures of the CNA profiles of the CRLM were analyzed for prognostic associations among patients with MSS cancers and R0/R1 resection: the genome‐wide CNA burden (*n* = 62, the mean across lesions for patients with multiple CRLM analyzed) and the intrapatient intermetastatic CNA heterogeneity estimate (*n* = 48). Both these patient‐wise CNA measures were categorized into a high and low group relative to the respective median in the patient series. High CNA heterogeneity or CNA burden was not overrepresented according to any of the clinical variables listed in Table 2. A high overall CNA burden was significantly associated with a poor 5y‐CSS rate in univariable analyses, with survival rates of 15% and 44% in the high and low groups, respectively (*P* = 0.02; Fig. 5). CNA burden, measured as the fraction of the genome affected by copy number aberrations, was also significantly associated with a poor patient outcome when analyzed as a continuous variable (HR 1.03, 95% CI 1.01–1.05, *P* = 0.009). Furthermore, patients with high intrapatient intermetastatic CNA heterogeneity also had a poorer survival rate than patients with a low heterogeneity, although not statistically significant in this smaller patient subgroup (5y‐CSS of 23% and 37%, respectively, *P* = 0.2; Fig. 5). The combination of a high CNA burden and a high CNA heterogeneity was associated with a particularly poor patient outcome, and patients in this subgroup had a 5yr‐CSS rate of 9%, compared to 30% among patients with only one of the variables high and 50% among patients low for both CNA measures (*P* = 0.02, test for trend; Fig. 5). The median survival rates in the three groups were 25, 36, and 50 months, respectively.

### 3.5 Combined biomarker analyses suggest potential for stratification of the *RAS/BRAF* ^V600E^ */TP53‐*mutated subgroup by CNA profiles

**Fig. 6:**
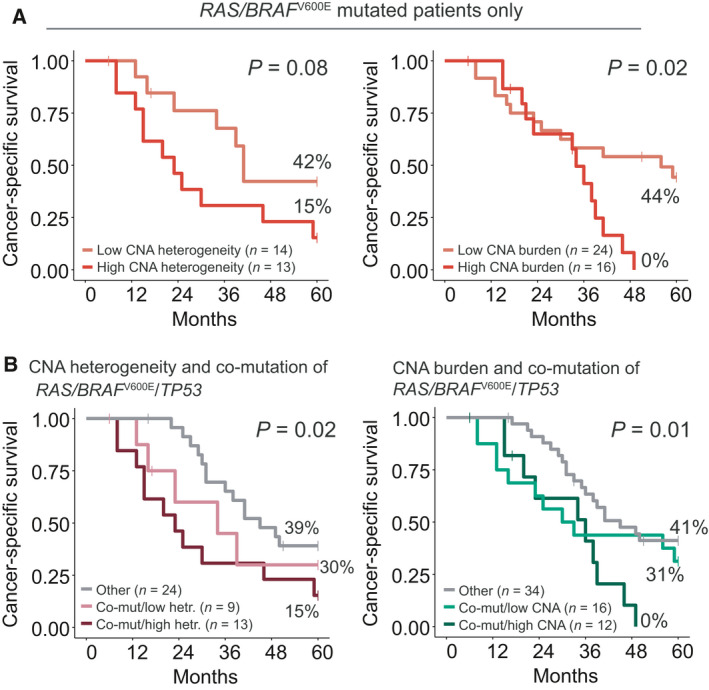
(A) The *RAS/BRAF* ^V600E^ *‐*mutated patient subgroup stratified by CNA heterogeneity (*n* = 27; left) and CNA burden (*n* = 40; right). (B) Patients with co‐mutated *RAS/BRAF* ^V600E^/TP53 stratified according to CNA heterogeneity (*n* = 46; left) and CNA burden (*n* = 62; right). *P* values are derived from log rank tests for comparisons of two groups and log rank tests for trend for comparisons of more than two groups.

Both CNA heterogeneity and CNA burden were significantly higher in patients with *TP53*‐mutated compared to wild‐type tumors, but the CNA estimates were not associated with *RAS*/*BRAF* ^V600E^ mutation status. Furthermore, there was a substantial variation in the CNA estimates within the mutational subgroups (Fig. 3B), motivating us to analyze the different prognostic biomarkers individually and combined. Within the *RAS*/*BRAF* ^V600E^ *‐*mutated subgroup, the 5y‐CSS was 15% in patients with a high level of intermetastatic CNA heterogeneity versus 42% in patients with low CNA heterogeneity (*P* = 0.08). Similarly, the 5y CSS was 0% in the *RAS*/*BRAF* ^V600E^ *‐*mutated patients with a high CNA burden versus 44% in *RAS*/*BRAF* ^V600E^‐mutated patients with a low CNA burden (*P* = 0.02; Fig. 6A). Prognostic stratification of the *TP53*‐mutated subgroup by either of the CNA estimates was not statistically significant (*P* ≥ 0.2; Fig. S5b). The triple combination of co‐mutation in *RAS*/*BRAF* ^V600E^ and *TP53* and high intermetastatic CNA heterogeneity was associated with a worse 5y‐CSS compared with co‐mutations/low heterogeneity and the remaining patients (*P* = 0.02 for analysis of trend among the three groups; Fig. 6B). Similar stratification of patients with co‐mutations by the CNA burden showed a prognostic association also for patients with a triple combination of co‐mutations and high CNA burden (*P* = 0.01, test for trend; Fig. 6B). Both associations were also supported by univariable Cox regression analyses (Table 3). Similar results were found when excluding patients with extrahepatic metastases from the analyses (Fig. S5c).

## 4 Discussion

Intrapatient molecular heterogeneity is anticipated to have clinical implications [35], and current evidence in metastatic CRC suggests that heterogeneity on the DNA copy number level is more widespread than heterogeneity of single nucleotide variants (SNVs) and small insertions/deletions (indels), at least in cancer‐critical genes [23, 25, 26]. We have shown that mutations in *KRAS, NRAS, BRAF* ^V600E^ [19], and *TP53* are predominantly homogeneously present among multiple resected CRLM from each patient. The DNA copy number states of the four genes were more heterogeneous among metastases and correlated with the genome‐wide intermetastatic CNA heterogeneity, consistent with a lower selection pressure for these genes on the DNA copy number level than on the point mutation level. Furthermore, high‐level amplifications targeting cancer‐critical genes, including the therapeutic targets *ERBB2* and *EGFR*, were also typically homogeneously present within patients, both among multiple metastatic lesions and in the primary tumor. The timing of cancer‐critical amplifications is poorly studied in CRC, and our results suggest that driver amplicons commonly arise before metastatic dissemination. In contrast, the level of genome‐wide intermetastatic DNA copy number heterogeneity beyond amplification events varied substantially among patients. There was no enrichment or depletion of cancer‐related genes among genomic regions with heterogeneous DNA copy number, suggesting that CNA heterogeneity is a genome‐wide and target‐ignorant characteristic.

There is an urgent clinical need for markers to identify patients with resectable or potentially resectable CRLM who are likely to have a long‐term benefit from surgery and systemic perioperative treatment. Analysis of circulating tumor DNA has demonstrated strong potential in the adjuvant or nonresectable settings, for detection of minimal residual disease and monitoring of response to systemic therapy [36]. Such noninvasive testing of prognostic markers prior to surgery is currently limited, although a trend for a prognostic effect of *KRAS* mutations in preoperative ctDNA was seen in a recent study [37]. *BRAF* ^V600E^ and *RAS* mutations are the molecular markers with best documented prognostic value, but their use in selection of patients for hepatectomy is currently not supported. *BRAF* ^V600E^ has been shown to have the strongest prognostic effect size, but a low prevalence of only 3–5% among patients with resectable CRLM [17], and < 2% in this study. *RAS* mutations identify a larger patient subgroup, but have weaker prognostic value, which suggests molecular heterogeneity among patients with *RAS*‐mutated cancers. In primary CRC, the prognostic value of *KRAS* has been suggested to be limited to MSS cancers and to depend on the consensus molecular subtypes [38]. In patients with resectable CRLM, the prognostic value may depend on co‐occurring *TP53* mutations [14, 15] or *TP53*/*SMAD4* mutations [16]. Our study supports the potential for improved prognostic stratification of patients with resectable CRLM based on *RAS*/*BRAF* ^V600E^ and *TP53* co‐mutations, although the study is not sufficiently powered to conclude on the independent prognostic value of individual mutations, in particular the low‐prevalence *BRAF* ^V600E^ and *NRAS* mutations. Another potential limitation of our study is the weaker sensitivity of Sanger sequencing than high‐throughput sequencing for mutation detection, although this concern was reduced by multiple sampling and the generally low level of tumor heterogeneity of CRC‐critical mutations.

We further suggest that high intermetastatic genomic heterogeneity confers poor outcome within the *RAS‐*mutated subgroup and show a potential for further prognostic stratification of the *RAS*/*BRAF* ^V600E^ and *TP53* co‐mutated subgroup by combined analyses with genome‐wide CNA profiles. Although CNA burden and the level of CNA heterogeneity were independent of *RAS* mutation status, patients with *TP53‐*mutated tumors had more extensive intermetastatic CNA heterogeneity and a higher CNA burden than patients with wild‐type tumors, suggesting a confounding prognostic effect. Loss of normal *TP53* expression has previously been associated with tolerability to aneuploidy [39, 40, 41, 42, 43, 44, 45], and it is conceivable that *TP53* mutations are needed for a submissive state that allows extensive copy number heterogeneity to evolve. The CNA heterogeneity estimate had nonsignificant prognostic associations, while a high CNA burden was significantly associated with poor cancer‐specific survival. The latter is in line with a recent pan‐cancer study of metastatic disease [46]. Our study cannot conclude on the independent prognostic value of CNA heterogeneity and *TP53* mutations in patients with *RAS‐*mutated CRLM, although there was a significant trend for poorer patient survival in the *RAS/BRAF* ^V600E^ */TP53* co‐mutated/high CNA heterogeneity group versus co‐mutated/low heterogeneity versus remaining patients. In accordance with a recent report [14], multivariable analysis with clinicopathological variables supports the independent poor‐prognostic associations of co‐mutated *RAS*/*BRAF* ^V600E^ and *TP53* CRLMs.

It has been debated whether the association between residual disease and outcome may reflect underlying cancer biology, as mutated *RAS* is associated with both a positive resection margin and early development of lung metastases [10, 11, 47]. However, excluding the patients with extra‐hepatic metastases did not impact on the prognostic associations found in this study.

## 5 Conclusions

We have described genomic heterogeneity on the DNA copy number level in patients with resectable CRLM, also within patient subgroups defined by *RAS*/*BRAF* ^V600E^ and *TP53* mutations. By combined biomarker analyses, we support the superior prognostic value of *RAS*/*BRAF* ^V600E^ and *TP53* co‐mutations compared with either mutation alone. Furthermore, a high level of intrapatient intermetastatic CNA heterogeneity or CNA burden may identify a subgroup of *RAS*/*BRAF* ^V600E^/*TP53‐*mutated cancers associated with a particularly poor outcome.

## Conflict of interest

The authors declare no conflict of interest.

## Author contributions

KCGB, AS, AN, and RAL involved in study concept and design; all authors performed the acquisition of data; KCGB, THB, AS, AN, and RAL performed the analysis and interpretation of data; KCGB, THB, AS, and RAL drafted the manuscript; all authors involved in critical revision and approval of the final manuscript; AN and RAL supervised the study.

### Peer Review

The peer review history for this article is available at https://publons.com/publon/10.1002/mol2.12885.

## Supporting information


**Fig. S1.** a) An alternative pipeline for estimation of CNA heterogeneity was tested, where the CNA heterogeneity score was calculated based on data segmented by the PCF algorithm from the R copy number package, including only segments with variance > 0.3 per comparison, similar to Sveen et al. 2016. The heterogeneity measures derived from the alternative pipeline (x‐axis) and that from the main analysis, using the ASCAT algorithm (y‐axis) were correlated. b) The copy number states for *KRAS*, *NRAS*, *BRAF* ^V600E^ and *TP53* were heterogeneous across samples. The four panels show the number of additional copies of the four genes in 176 metastatic lesions from 48 patients, sorted patient‐wise and grouped according to the mutation statuses of the two genes. The gray bars below the heatmaps denotes the change from one patient to the next. d) Heterogenous copy number states for *KRAS*, *NRAS*, *BRAF* ^V600E^ and *TP53* reflected the genome‐wide CNA heterogeneity score, with a higher genome‐wide heterogeneity scores in patients where the particular genes had intermetastatic heterogeneous copy number states.

**Fig. S2.** a) Overview of intrapatient concordance of the 35 amplification events in 19 patients. Each count (y‐axis) is a unique amplification event in one patient. The x‐axis shows the fraction of the metastases from the given patient with concordant amplification. For example, a fraction of 0.5 indicates that half of the metastases from the patient in question have concordant amplification, while a fraction of 1 indicates that all metastases from the given patient have concordant amplification. Thirty‐one per cent of the amplification events were fully concordant at a ≥ 15 additional copies level (i.e., all the metastatic lesions from the given patient had ≥ 15 additional copies), a threshold of 5 additional copies to accept concordance resulted in 69% intrapatient concordance. b) For the 12 amplification events affecting cancer‐critical genes, 50% were concordant at ≥ 15 additional copies in all lesions from the affected patient, while a threshold of 5 additional copies to accept concordance resulted in 75% intrapatient concordance.

**Fig. S3.** Summarized frequencies of DNA copy number aberrations across 64 patients (192 lesions). For patients with more than one lesion available, the frequencies were summarized per patient by calling gains and losses in any given genomic region when they occurred in at least one lesion from that patient. In cases where at least one lesion had gain while at least one lesion had loss in the same genomic region, both a gain and a loss in this region was called.

**Fig. S4.** Heterogeneity measures based on either Euclidean distance, correlation‐based distance or fraction of discordant CNAs were highly concordant irrespective of whether they were estimated based on a genome‐wide approach or based on cancer‐critical genes only (Spearman’s rho ≥0.93). Also, the heterogeneity estimates from the three different methods were correlated to one another (Spearman’s rho ≥0.63).

**Fig. S5.** a) *RAS* mutations and *RAS*/*TP53* co‐mutations were persistently associated with poor patient outcome when excluding patients with *BRAF* ^V600E^ mutations from the analysis. b) A high CNA heterogeneity or CNA burden did not significantly stratify patients with *TP53* mutated tumors according to patient outcome. d) A high CNA heterogeneity and CNA burden still stratified patients with *RAS*/*BRAF* ^V600E^ and *TP53* co‐mutated tumors in terms of outcome when patients with extrahepatic metastases where excluded from the analysis, although nonsignificantly for CNA burden. P values are derived from log rank tests for comparisons of two groups and log rank tests for trend for comparisons of more than two groups.

**Table S1.** Primers for Sanger sequencing.
**Table S2.** Correlation between CNA heterogeneity score (calculated as the intrapatient mean pairwise Euclidean distance) and other CNA variables.
**Table S3.** Overrepresentation of *RAS*/*BRAF* ^V600E^ and *TP53* co‐mutation according to key clinicopathological variables (n = 171 patients).

## Data Availability

The datasets supporting the conclusions of this article can be obtained from the authors upon reasonable request.
