# Supplementary figures and images for: Genomic and prognostic heterogeneity among RAS/BRAF V600E/TP53 co‐mutated resectable colorectal liver metastases
Source: Mol Oncol. 2021 Jan 8;15(4):830–45. doi: 10.1002/1878-0261.12885 (PMC8024718; doi:10.1002/1878-0261.12885)

# Supplementary Figure 1

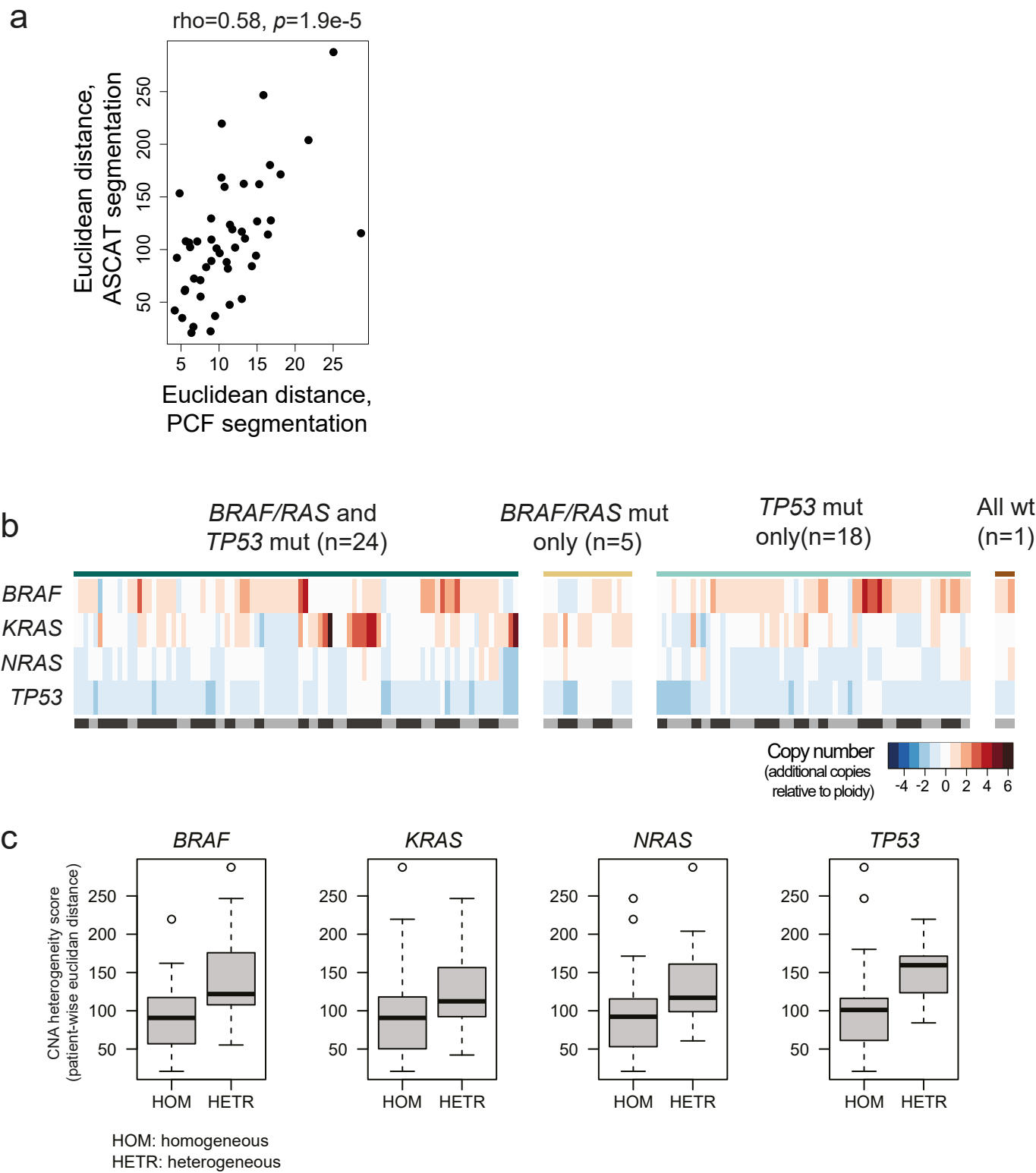

Supplement: Supplementary file 1 — Fig. S1. a) An alternative pipeline for estimation of CNA heterogeneity was tested, where the CNA heterogeneity score was calculated based on data segmented by the PCF algorithm from the R copy number package, including only segments with variance > 0.3 per comparison, similar to Sveen et al. 2016. The heterogeneity measures derived from the alternative pipeline (x‐axis) and that from the main analysis, using the ASCAT algorithm (y‐axis) were correlated. b) The copy number states for KRAS, NRAS, BRAF V600E and TP53 were heterogeneous across samples. The four panels show the number of additional copies of the four genes in 176 metastatic lesions from 48 patients, sorted patient‐wise and grouped according to the mutation statuses of the two genes. The gray bars below the heatmaps denotes the change from one patient to the next. d) Heterogenous copy number states for KRAS, NRAS, BRAF V600E and TP53 reflected the genome‐wide CNA heterogeneity score, with a higher genome‐wide heterogeneity scores in patients where the particular genes had intermetastatic heterogeneous copy number states. [file MOL2-15-830-s003.pdf]

# Supplementary Figure 3

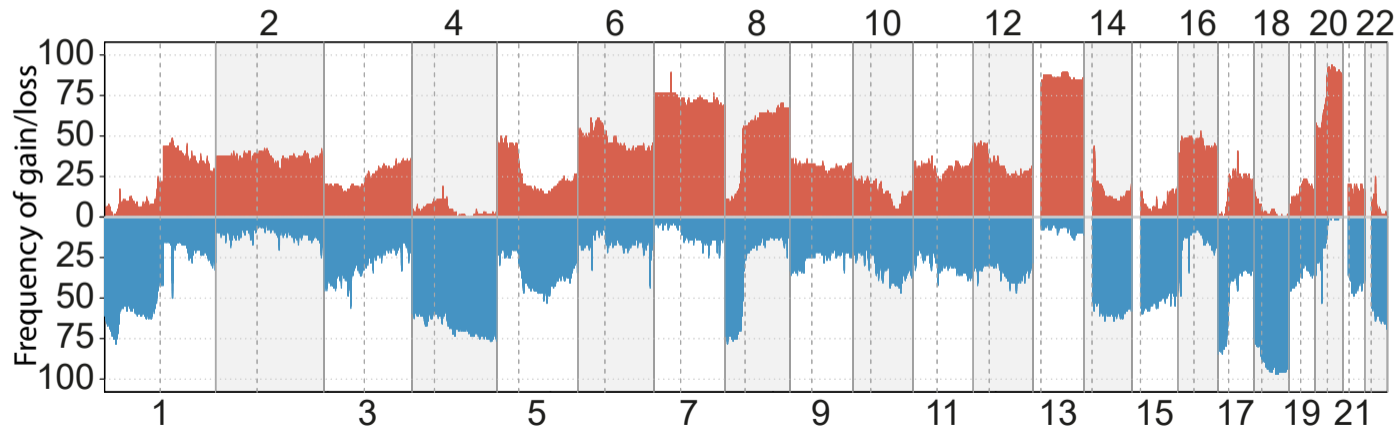

Supplement: Supplementary file 3 — Fig. S3. Summarized frequencies of DNA copy number aberrations across 64 patients (192 lesions). For patients with more than one lesion available, the frequencies were summarized per patient by calling gains and losses in any given genomic region when they occurred in at least one lesion from that patient. In cases where at least one lesion had gain while at least one lesion had loss in the same genomic region, both a gain and a loss in this region was called. [file MOL2-15-830-s002.pdf]

# Supplementary Figure 4

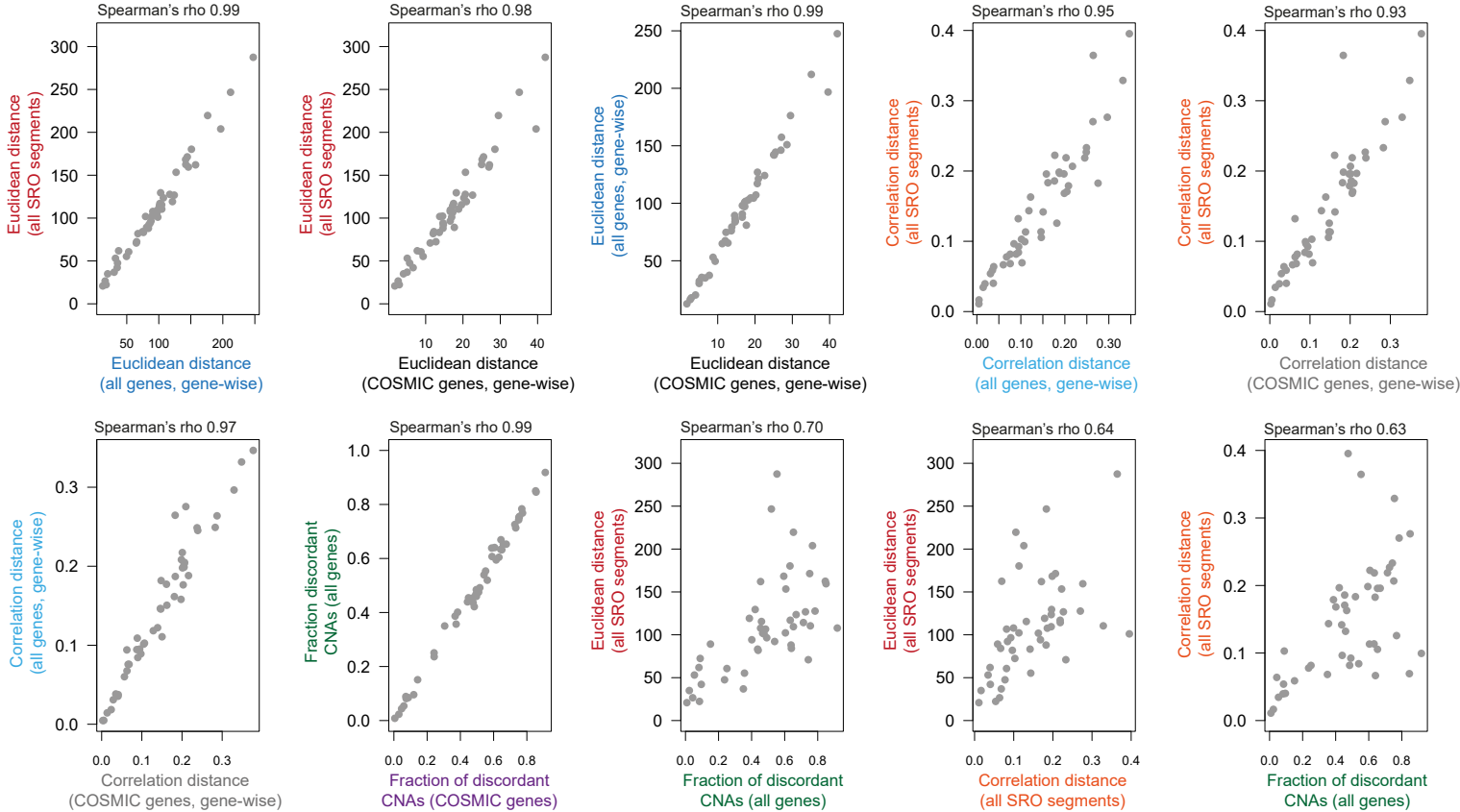

Supplement: Supplementary file 4 — Fig. S4. Heterogeneity measures based on either Euclidean distance, correlation‐based distance or fraction of discordant CNAs were highly concordant irrespective of whether they were estimated based on a genome‐wide approach or based on cancer‐critical genes only (Spearman’s rho ≥0.93). Also, the heterogeneity estimates from the three different methods were correlated to one another (Spearman’s rho ≥0.63). [file MOL2-15-830-s001.pdf]

# Supplementary Figure 5

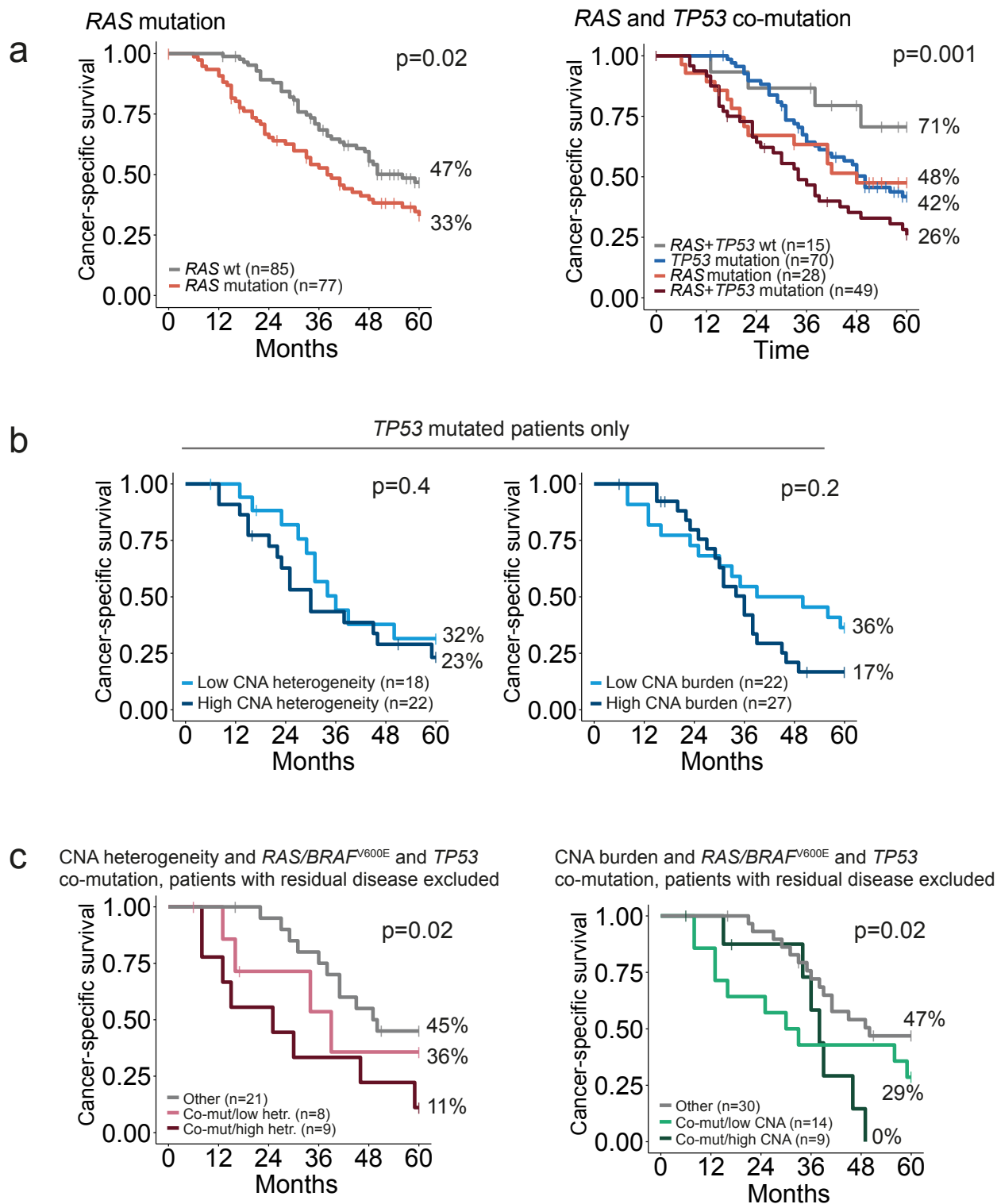

Supplement: Supplementary file 5 — Fig. S5. a) RAS mutations and RAS/TP53 co‐mutations were persistently associated with poor patient outcome when excluding patients with BRAF V600E mutations from the analysis. b) A high CNA heterogeneity or CNA burden did not significantly stratify patients with TP53 mutated tumors according to patient outcome. d) A high CNA heterogeneity and CNA burden still stratified patients with RAS/BRAF V600E and TP53 co‐mutated tumors in terms of outcome when patients with extrahepatic metastases where excluded from the analysis, although nonsignificantly for CNA burden. P values are derived from log rank tests for comparisons of two groups and log rank tests for trend for comparisons of more than two groups. [file MOL2-15-830-s004.pdf]
