# Supplementary material for: Genomic and prognostic heterogeneity among RAS/BRAF V600E/TP53 co‐mutated resectable colorectal liver metastases
Source: Mol Oncol. 2021 Jan 8;15(4):830–45. doi: 10.1002/1878-0261.12885 (PMC8024718; doi:10.1002/1878-0261.12885)

Supplementary Figure 2

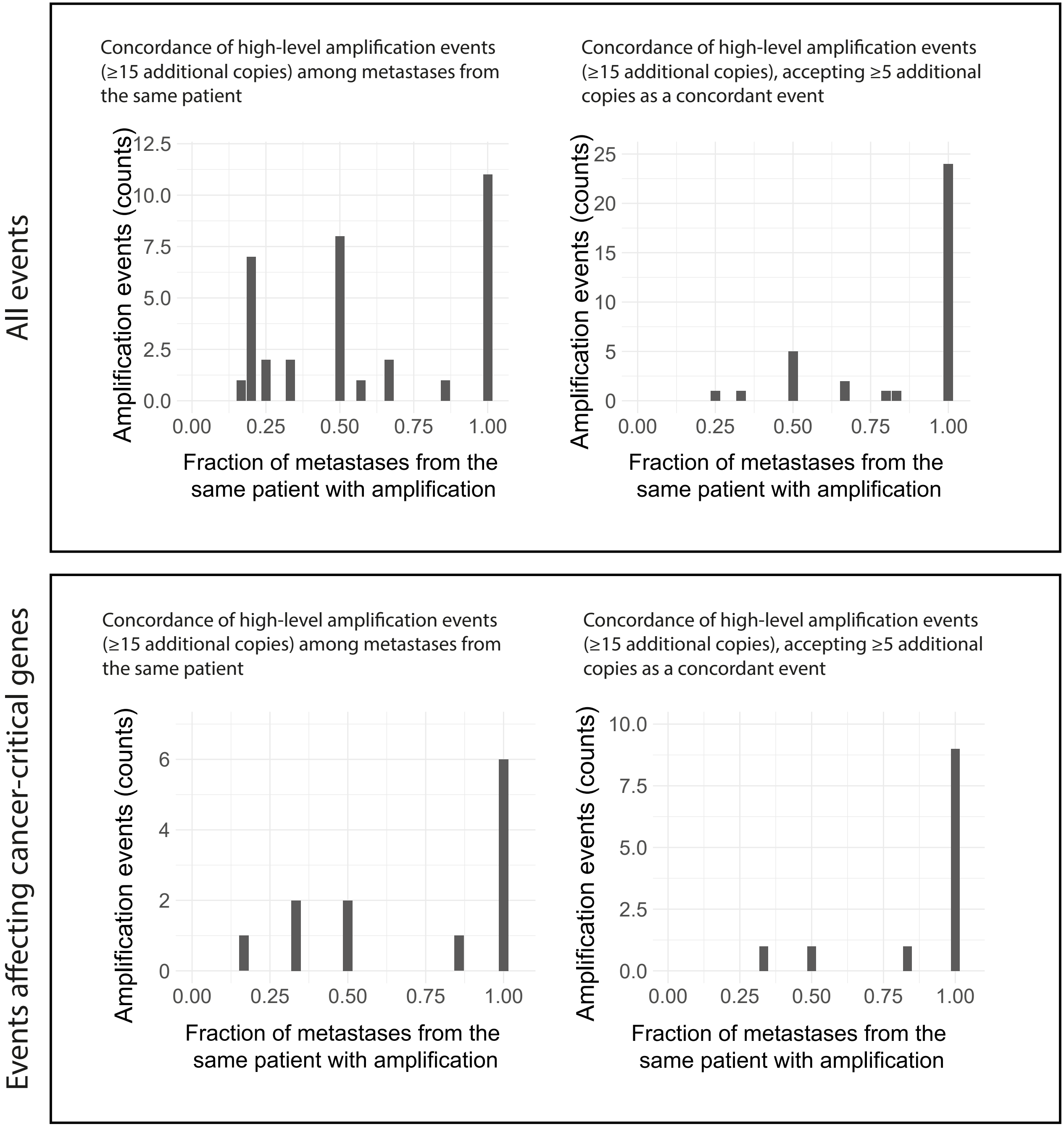

Supplement: Supplementary file 2 — Fig. S2. a) Overview of intrapatient concordance of the 35 amplification events in 19 patients. Each count (y‐axis) is a unique amplification event in one patient. The x‐axis shows the fraction of the metastases from the given patient with concordant amplification. For example, a fraction of 0.5 indicates that half of the metastases from the patient in question have concordant amplification, while a fraction of 1 indicates that all metastases from the given patient have concordant amplification. Thirty‐one per cent of the amplification events were fully concordant at a ≥ 15 additional copies level (i.e., all the metastatic lesions from the given patient had ≥ 15 additional copies), a threshold of 5 additional copies to accept concordance resulted in 69% intrapatient concordance. b) For the 12 amplification events affecting cancer‐critical genes, 50% were concordant at ≥ 15 additional copies in all lesions from the affected patient, while a threshold of 5 additional copies to accept concordance resulted in 75% intrapatient concordance. [file MOL2-15-830-s005.pdf]
